# Laparoscopic cholecystectomy after percutaneous transhepatic gallbladder drainage for acute cholecystitis in a patient with a left ventricle assist device: A case report and brief review of the literature (with video)

**DOI:** 10.1002/ccr3.5800

**Published:** 2022-05-22

**Authors:** Takehiko Hanaki, Teppei Sunaguchi, Keisuke Goto, Masaki Morimoto, Yuki Murakami, Naruo Tokuyasu, Shuichi Takano, Teruhisa Sakamoto, Toshimichi Hasegawa, Yoshiyuki Fujiwara

**Affiliations:** ^1^ Department of Gastrointestinal and Pediatric Surgery School of Medicine Tottori University Faculty of Medicine Yonago Japan

**Keywords:** acute cholecystitis, laparoscopic cholecystectomy, left ventricular assist device, percutaneous transhepatic gallbladder drainage

## Abstract

For acute cholecystitis in patients with left ventricular assist devices, the use of percutaneous transhepatic gallbladder drainage to calm inflammation before planned laparoscopic cholecystectomy may be helpful in safely adjusting anticoagulation and in performing safe laparoscopic cholecystectomy, as demonstrated in this case.

## Graphical abstract

When acute cholecystitis is encountered in a patient with a left ventricular assist device, performing laparoscopic cholecystectomy (LC) after reducing inflammation with PTGBD, instead of performing an emergency LC may be useful.

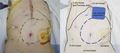

## 1 INTRODUCTION

To facilitate blood circulation in the failing heart, the left ventricle assist device (LVAD) was developed and used as a bridge to transplant or destination therapy. The LVAD is an alternative to heart transplantation and can be a bridge to recovery or a bridge to heart transplantation^1^, ^2^, ^3^ in patients with left ventricular failure. The use of LVADs prolongs survival in patients with heart failure.^3^ In Japan, LVADs became a health insurance option for patients on the heart transplant waiting list in 2011. However, the number of patients on the transplant waiting list and, consequently, the number of patients with LVADs is increasing due to the continuing shortage of donor hearts.^3^, ^4^, ^5^ Adequate anticoagulation is essential in patients with LVADs, as clots inside the LVAD can be fatal. However, anticoagulation can lead to intraoperative and postoperative bleeding when patients with LVADs require surgery.

Acute cholecystitis is a common abdominal condition that requires urgent intervention. Several reports describe the use of endoscopic surgery to treat cholecystitis in patients with LVADs.^6^, ^7^ Here, we report the use of percutaneous transhepatic gallbladder drainage (PTGBD) to relieve local inflammation due to acute cholecystitis in a LVAD transplant recipient with unstable vital status. The PTGBD was followed by an elective laparoscopic cholecystectomy (LC). An intraoperative video is provided (Video S1).

## 2 CASE REPORT

### 2.1 Case presentation

A 64‐year‐old man on the heart transplant waiting list was admitted to the cardiovascular surgery ward of our hospital for intracerebral hemorrhage. He had undergone LVAD (HeartMate II, Thoratec Corporation) implantation at 59 years of age due to dilated cardiomyopathy causing left ventricle failure. The subcortical hemorrhage was treated conservatively, and the patient was rehabilitated in the same ward without any complications. However, the patient developed fever, nausea, and right upper quadrant pain with shock vitals (blood pressure, 82/72 mmHg; heart rate, 110 beats/min; body temperature, 38.2°C). Computed tomography revealed an enlarged gallbladder with a thickened wall and surrounding fatty tissue opacity. He was diagnosed with acute cholecystitis and was referred to our department. Given that the patient's performance status was poor, and he was judged to have grade III (severe) cholecystitis^8^ with organ failure [shock vitals with heart failure requiring LVAD requiring noradrenaline (0.05 μg/kg/min)], we decided to perform PTGBD to reduce inflammation in the gallbladder. The prothrombin time‐international normalized ratio (PT‐INR) was 4.33; therefore, following the administration of 2000 IU of prothrombin complex concentrate (35 IU/kg), PTGBD was performed after confirming that the PT‐INR recovered to 1.44. Gallbladder drainage was performed by the usual percutaneous transhepatic route with puncture of the right hypochondriac lesion using ultrasonography. Catecholamine could be discontinued the day after the surgery, and the patient was treated with antibiotics (cefmetazole 3 g/day) for about 2 weeks. The cholecystitis resolved within a few days after PTGBD. An elective LC was planned for 3 months after the PTGBD, to avoid the risk of recurrent cholecystitis that could cause LVAD infection.

**FIGURE 1:**
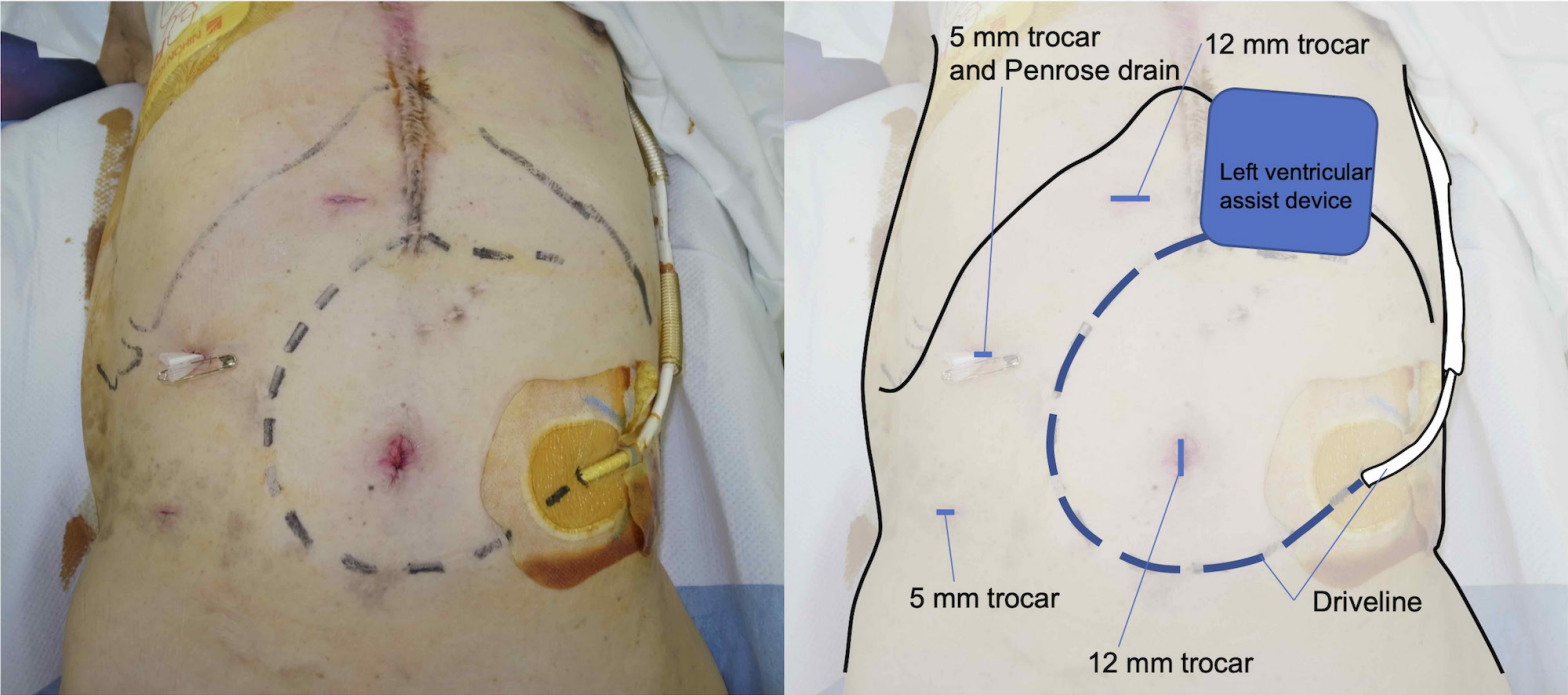
Postoperative abdominal photograph showing the port placement and the position of the subcutaneous driveline markings

**FIGURE 2:**
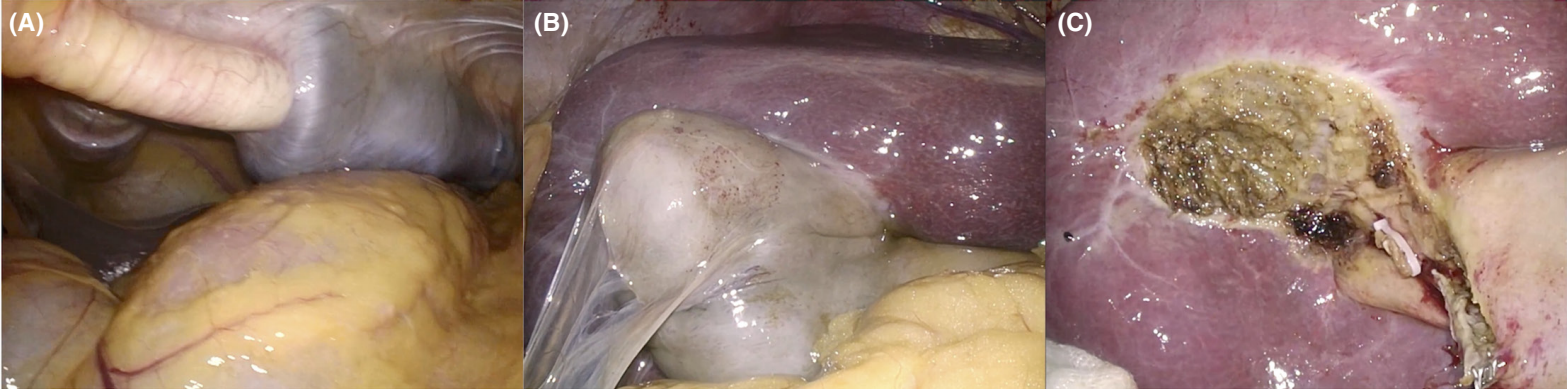
Intraoperative pictures showing the left ventricle assist device. (A) The left subcostal preperitoneal space, (B) adhesion around the gallbladder, and (C) the liver bed after cholecystectomy

The patient was taking 3.5 mg/day of warfarin with a PT‐INR of 2.0–3.0 because of the LVAD. In preparation for surgery, the anticoagulation was reversed with prothrombin complex concentrate (2000 IU/body, 35 IU/kg). After the induction of general anesthesia, markings were made on the skin along the subcutaneous driveline to avoid injury during trocar insertion (Figure 1). The first endoscopic trocar was inserted via the open method (15 mm vertical incision at the umbilicus). The pneumoperitoneum pressure was set at 10 mmHg and a 30° scope was inserted. The other trocars were placed as demonstrated in Figure 1. No adhesions were found around the LVAD, which was placed in the preperitoneal space. However, some adhesions were found around the gallbladder (Figure 2A,B, Video S1). The neck of the gallbladder was thickened, and a sub‐total resection was performed. To ensure that there was no postoperative bleeding, a drain was placed in the liver bed, and the operation was completed. The duration of surgery was 114 min, and the estimated amount of blood loss was 5 ml.

### 2.2 Postoperative clinical course

**FIGURE 3:**
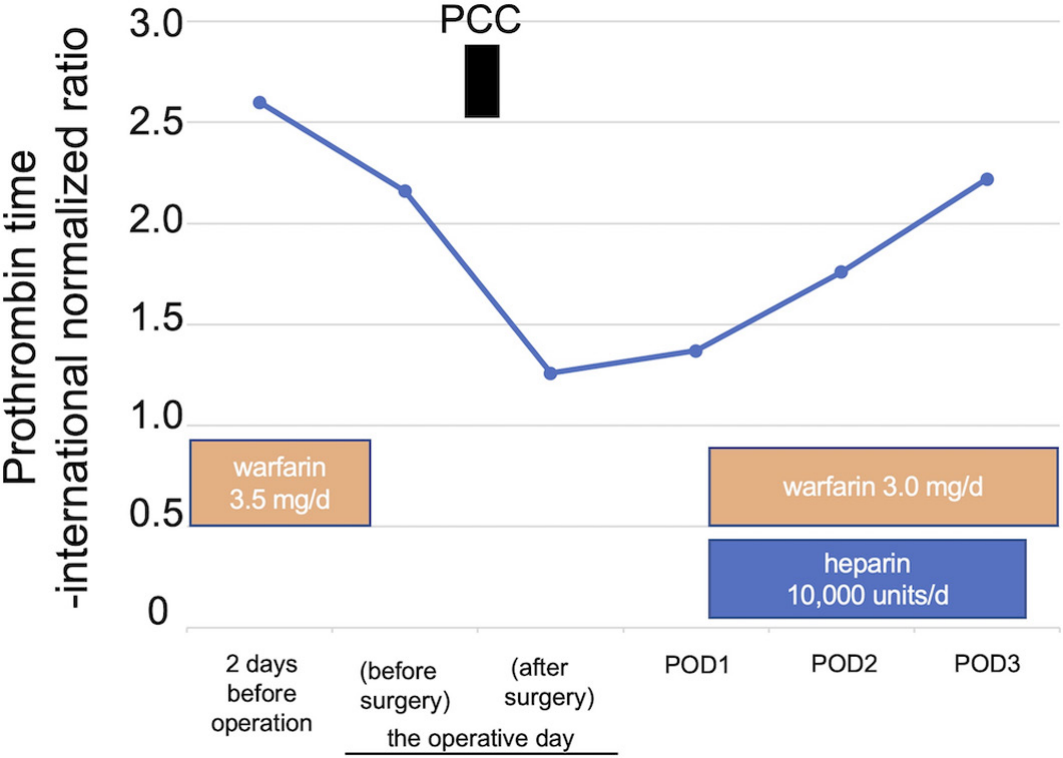
Perioperative anticoagulation management. PCC, prothrombin complex concentrate; POD, postoperative day

The patient was returned to the intensive care unit, and anticoagulants were restarted on postoperative day (POD) 1 with 10,000 units/d of heparin and 3 mg/d of warfarin. On POD3, prolonged coagulation was observed, and heparin was terminated (Figure 3). The patient was transferred from the intensive care unit to the cardiovascular surgery ward on the same day without using cardiovascular drugs. The postoperative course was uneventful. No LVAD‐related problems occurred, and no postoperative blood transfusion was required. 11 months after the LC, the patient is on the waiting list for a heart transplant.

## 3 DISCUSSION

According to our experience and previous reports, the laparoscopic approach to cholecystectomy may be useful in patients with LVADs.^6^ Pneumoperitoneum may increase the preload on the heart and cause vital changes in LVAD patients.^9^ However, in this case, no intraoperative change due to pneumoperitoneum was observed. In the present study, we performed pneumoperitoneum at 10 mmHg. However, previous reports suggest that a pneumoperitoneum at 10–15 mmHg is also acceptable (Table 1).^6^, ^10^, ^11^, ^12^, ^13^, ^14^, ^15^, ^16^, ^17^, ^18^, ^19^, ^20^, ^21^, ^22^, ^23^, ^24^

**TABLE 1:** Reports of laparoscopic cholecystectomy in patients with left ventricular assist devices

Year	Authors	Number of cases	Age	Sex	VAD type	Type of surgery	Pressure of pneumoperitoneum (mmHg)	Bleed (ml)	Operation time (min)	Conversion	Complication grade (Clavien‐Dindo classification)	PTGBD
1994	Votap^10^	1	19	n.d.	Thoratec VAD system	n.d.	n.d.	n.d.	n.d.	n.d.	IIIb (reoperation for hemostasis)	n.d.
2004	Eckhaus^11^	1	68	Male	Novacor	Elective	n.d.	n.d.	n.d.	No	II (driveline infection)	No
2005	Nissen^12^	1	54	Male	Thoratec VAD system	Emergent	15	n.d.	45	No	IIIb (relaparoscopy for hemostasis)	No
2008	Kartha^13^	1	51	Male	HeartMateII	Elective	15	n.d.	n.d.	No	0	No
2009	Livi^14^	1	47	Male	Exocor	Emergent	12	n.d.	n.d.	No	0	No
2012	Amir^15^	1	40	Male	HeartMateII	Emergent	n.d.	n.d.	25	No	0	No
2013	Naitoh^6^	1	31	Male	DuraHeart	Emergent	10	430	160	No	0	No
2014	Eck^16^	2	40	Male	HeartMateII	Emergent	n.d.	n.d.	n.d.	No	0	No
			68	Male	HeartMateII	Elective	n.d.	n.d.	n.d.	No	n.d.	Yes
2015	Reich^17^	7	n.d.	n.d.	n.d.	n.d.	12–15	n.d.	n.d.	No	n.d.	n.d.
2015	Yoon^18^	1	53	Male	HeartMateII	Elective	n.d.	n.d.	n.d.	No	0	No
2016	Ashfaq^19^	6	63 ± 17	5 males, 1 female	4 HeartMateII/1 CentriMAG/1 CardioWest	n.d.	n.d.	n.d.	n.d.	No	(Acute kidney injury in one patient, pneumonia in one patient)	n.d.
2018	Suresh^20^	5	67 ± 4.4	5 males	n.d.	n.d.	n.d.	n.d.	n.d.	n.d.	(Abdominal wall hematoma in one patient)	n.d.
2019	Vigneswaran^21^	6	n.d.	n.d.	HeartMateII/HeartWare	Elective	n.d.	130±184	138±66	No	n.d.	n.d.
2019	Takagi^22^	1	56	Male	Jarvik 2000	Emergent	10	240	119	No	0	No
2020	Zibermints^23^	2	63	Male	HeartMateII/HeartWare	Elective	n.d.	n.d.	n.d.	Yes	II	n.d.
			66	Male		Elective				No	0	n.d.
2020	Beetz^24^	4	n.d.	n.d.	n.d.	Elective	n.d.	n.d.	n.d.	n.d.	(Blood transfusion in three patients)	n.d.
2022	Present Case	1	64	Male	HeartMateII	Elective	10	5	114	No	0	Yes

Note

VAD, ventricular assist device; Thoratec VAD system, Thoratec Laboratories, Pleasanton, CA; Novacor, WorldHeart, Oakland, CA; HeartMateII, Abbott, Plymouth, MN; Exocor, Berlin Heart GmbH, Berlin, Germany; CentriMag, Thoratec Laboratories, Pleasanton, CA; CardioWest, SynCardia Systems, Tucson, AZ; HeartWare, Medtronic, Dublin, Ireland; Jarvik2000, JarvikHeart, New York, NY.

Abbreviations: PTGBD, percutaneous transhepatic gallbladder drainage; n.d., not described.

There are currently nine case reports and seven case series in the literature, including 32 cases that described LC (Table 1). Of the 42 total cases (including the presented case), only one case (2.4%) converted to open surgery. Nearly half of the patients (40.5%) underwent scheduled elective surgery. This trend seems to be particularly strong since 2015. We speculate that this change is partly related to increased awareness of the acute cholecystitis guidelines,^8^ that is, the use of alternative methods such as gallbladder drainage rather than emergency surgery in the case of organ failure. However, the rate of PTGBD was not stated in many of the previous reports; therefore, the overall rate is unknown. The safety and efficacy of PTGBD have been confirmed by many case‐control studies in patients with cholecystitis. PTGBD is recommended as a standard drainage method for patients with acute cholecystitis at high surgical risk.^8^ In this case, the following factors contributed to our success. First, the acute cholecystitis was treated with PTGBD to reduce inflammation before the scheduled surgery. Second, time was given before the scheduled surgery to consider warfarin antagonism. The fact that coagulant reversal was confirmed immediately before surgery also contributed to safe surgery. Third, the surgery was performed in a calm circulatory state. For acute cholecystitis in patients with LVADs, laparoscopic cholecystectomy after calming inflammation with PTGBD, rather than immediate emergency surgery even when vitals are stable, may be useful for safe patient management. In our case, the operation was completed safely, with lower blood loss compared with the blood loss in previous reports.

Although our institution is not a heart transplant hospital, LVAD transplantation is performed in the Department of Cardiovascular Surgery at Tottori University Hospital. Patients with LVADs are admitted to our hospital. As the number of patients with LVADs is increasing worldwide, more cases of acute abdominal problems in patients after LVAD transplantation will be encountered. The use of PTGBD to reduce cholecystitis before performing LC is beneficial in patients with LVADs.

In conclusion, we described the case of a patient with end‐stage heart failure and an implanted LVAD who was diagnosed with acute cholecystitis. The patient was successfully and safely treated with PTGBD followed by elective LC. In patients with LVADs who develop acute cholecystitis, the use of early PTGBD to eliminate inflammation in the biliary tract is useful. Awaiting surgery prevents fatal complications, such as perioperative bleeding and biliary tract injury.

## CONFLICT OF INTEREST

The authors have no conflicts of interest or financial ties to disclose.

## AUTHOR CONTRIBUTIONS

TaH collected the patient data, performed surgery, and a literature review, and wrote the manuscript. TS, KG, MM, YM, NT, ST, and TS revised the manuscript. ToH and YF were involved in the overall supervision of the study. All authors have read and approved the final version of the manuscript.

## CONSENT

Written informed consent was obtained from the patient for the publication of this case report and accompanying images. A copy of written consent is available for review by the Editor‐in‐Chief of this journal.

## Supporting information

Video S1

## Data Availability

Relevant data and images related to the patient's course and care are included in the article.
